# Challenges for the Newborn Following Influenza Virus Infection and Prospects for an Effective Vaccine

**DOI:** 10.3389/fimmu.2020.568651

**Published:** 2020-09-16

**Authors:** Martha A. Alexander-Miller

**Affiliations:** Department of Microbiology and Immunology, Wake Forest School of Medicine, Winston-Salem, NC, United States

**Keywords:** newborn, vaccine, influenza, antibody, adjuvant

## Abstract

Newborns are at significantly increased risk of severe disease following infection with influenza virus. This is the collective result of their naïve status, altered immune responsiveness, and the lack of a vaccine that is effective in these individuals. Numerous studies have revealed impairments in both the innate and adaptive arms of the immune system of newborns. The consequence of these alterations is a quantitative and qualitative decrease in both antibody and T cell responses. This review summarizes the hurdles newborns experience in mounting an effective response that can clear influenza virus and limit disease following infection. In addition, the challenges, as well as the opportunities, for developing vaccines that can elicit protective responses in these at risk individuals are discussed.

## Introduction

Infection with influenza virus places a large burden on human health. The WHO estimates there are 290,000–650,000 influenza-associated deaths and 3–5 million cases of severe disease globally each year (https://www.who.int/news-room/fact-sheets/detail/influenza-(seasonal)). In the U.S. alone, the CDC estimated 35.5 million people experienced influenza in the 2018–19 season (www.cdc.gov/flu/about/burden/2018-2019.html). Newborns and young infants represent a particularly susceptible population for severe disease following influenza virus infection. Even in countries like the U.S., where health care resources are widely available, those under the age of 6 months are six times more likely to die as a result of influenza virus infection compared to children between the ages of 13 and 17 years (1).

While it is known that infants experience a higher rate of infection than older children, a recent study from the Influenza and Respiratory Syncytial Virus in Infants (IRIS) study suggests that our current understanding of the rate of infection significantly underestimates the burden in infants (2). Analysis of 1,934 acutely ill, non-vaccinated infants (0–11 months) enrolled during the influenza season in four countries (Albania, Jordan, Nicaragua, and the Philippines) showed 254 (13%) were influenza virus positive by either PCR, serology, or both.

Disease states associated with influenza virus infection in infants and children include otitis media, pneumonia, myositis, and croup. The last is restricted primarily to individuals <1 year of age and can be life threatening. The risk of lower respiratory disease is significantly increased in children <2 years of age (3–7). In addition, bacterial pneumonia, which contributes to the increased lower respiratory disease in this age group, is a common complication of influenza virus infection (8). Bacterial coinfection has been shown to be a significant predictor of severe disease requiring admission to the pediatric intensive care unit (9). These data show that influenza virus continues to be a major health issue for newborns and young infants and establish the clear need for improved therapeutics and vaccines for this vulnerable population. Here, the underlying immune factors that contribute to the increased susceptibility to disease and the promise for the development of vaccines to protect these vulnerable individuals are discussed.

## The Infant Immune System

The increased susceptibility to severe disease in infants following influenza virus is a result of the naïve status of newborns together with the altered responsiveness of the immune system. Much of our current understanding of the neonatal immune response comes from studies performed in the mouse or from human cord blood cells, although there is a growing body of work in the non-human primate. The results of these studies reveal alterations across the immune system, impacting both the innate and adaptive arms of the response.

The innate response is the first line of defense against an invading pathogen. Monocytes and dendritic cells (DC) play critical roles in clearance of incoming virus as well as initiation of an adaptive immune response. Activation and recruitment of these cells is often regulated by pattern recognition receptor (PRR) signaling (10, 11). These pathogen sensing molecules promote the activation of multiple immune cell types and as such can be critical regulators of the early immune response to influenza virus infection (12).

Analysis of DC and monocytes isolated from human cord blood revealed that these cells are decreased in their capacity to respond to pathogen associated signals, e.g., TLR agonists, compared to cells isolated from adults (13–19). PRR engagement on newborn monocytes and DC differs from adult derived cells in that it often results in the induction of a robust anti-inflammatory (e.g., IL-10) coupled with a decreased pro-inflammatory response (20). In mouse models, the suboptimal responsiveness of DC from neonates manifests as a decrease in the expression of costimulatory molecules and a striking reduction in IL-12 (15–17), an important signal that promotes T helper 1 (Th1) differentiation. The impaired production of this important regulatory cytokine has been associated with increased T helper 2 (Th2) differentiation and poor Th1 responses (13), the latter of which is part of an optimal influenza virus-specific response. Th2 biased differentiation appears to be the result of expression of the IL-13Rα chain, which together with IL-4Rα can serve as an alternative receptor for IL-4 (21, 22).

The decreased activation/maturation of DC in response to stimulatory signals is compounded by alteration in the number/frequency of IL-12 producing APC available for surveillance. CD8^+^ and CD103^+^ DC are the major producers of IL-12 in mice (23, 24). The correlative population in humans, BDCA3^+^ DC, similarly produces high levels of IL-12 (25, 26). Newborn mice are reported to have a paucity of lymphoid tissue resident CD8^+^ DC (22), a DC subset that is an important contributor to the generation of a CD8^+^ T cell response. The alterations in DC extend to the tissue. Analysis of DC in the lungs of 1-week-old mice showed a reduced frequency of these populations as well as differences in DC subset distribution (27–29). Further, the ratio of CD103^+^ airway to CD11b^+^ parenchymal DC was reduced in neonatal mice (28), suggesting an even greater impact on this population that serves as a potent migratory APC for driving adaptive immune responses following influenza virus infection (30). In addition to the changes in number and distribution, DC in the lungs of newborn mice are impaired in both their ability to upregulate costimulatory molecules and in antigen processing following virus infection (29).

The distribution and quantity of DC subsets in LN and lung of human neonates has been minimally explored. In a study from dos Santos et al., airway DC were reported to be very rare in infants (31). In addition, few DC expressed DC-SIGN, a C-type lectin that plays a role in uptake and TLR signaling (31). Gaining a fuller understanding of the distribution and function of DC in the newborn lung will need to be an area of high priority as we strive to meet the challenge of developing new therapeutics to support immune function following influenza virus infection in this population.

Infant mice also have a highly reduced number of plasmacytoid DC (28). While not involved in T cell activation, these cells are important sources of type I IFN, which has anti-viral activity as well as being an important regulatory cytokine for adaptive responses. Although the role of these cells in adult mice was reported to be dispensable during influenza virus infection (32), their importance to the response in infants has not been evaluated.

NK cells are another important innate immune cell for the early control of influenza virus infection (33, 34). NK cells mediate killing of infected cells through multiple mechanisms including antibody dependent cellular cytotoxicity, direct release of cytotoxic granules, TRAIL, and FasL. They also produce a large array of cytokines such as IL-5, IL-10, IL-13, GM-CSF, TNFα, TGFβ, and IFNγ. Surprisingly perhaps, human neonates have comparable or higher numbers and percentages of NK cells in the peripheral blood compared to adults (35, 36). However, these cells have a less terminally differentiated phenotype, i.e., few express CD57 (37), a marker associated with high cytotoxic and low cytokine responses. NK cells in neonates also exhibit reduced levels of CD54, suggesting impaired adherence to target cells (38, 39) as well as increased expression of inhibitory receptors, e.g., NKG2a (40). Thus, although they are present, their ability to contribute to viral clearance is likely impaired. As with other cell types, we have a limited understanding of lung NK cell number and function in infants. In humans, NK cells appear to be present as they were identified in the epithelial layer of human infants who had died from causes not related to pulmonary disease (31). However, how this population seeds the lungs and their ability to function in infants has not been explored.

Compounding those present in the innate arm of the immune response, there are multiple challenges on the adaptive side of the house. Some of these are a result of the innate alterations described above, e.g., cytokine production or DC maturation; this is certainly a major contributor to the propensity for differentiation of CD4^+^ T cells into Th2 cells [for review see (41)]. Biased Th2 differentiation has also been reported in human cord blood cells (42) as well as in newborn non-human primates (NHP) (our unpublished data). However, in addition to the regulatory signals derived from accessory cells, there are inherent attributes of adaptive immune cells that exacerbate the challenges associated with mounting a response. T cells from human neonates exhibit a generalized defect in responsiveness and differentiation (41, 43–51), making it more difficult to activate T cells that do successfully engage antigen-bearing DC. Reported defects include reduced levels of the signaling molecules lck and ZAP-70 (48) as well as decreased AP-1 mediated transcription (52).

As if these hurdles were not high enough, we have an increasing appreciation for the heightened T regulatory cell (T_reg_) response that is present in newborns and young infants. Studies in human infants reveal a higher representation of Tregs in circulation (53–59), a finding also seen in NHP [(60) and manuscript *in press*]. This may be the result of the enhanced propensity for cells to differentiate into Tregs in these individuals (59, 61–63). The increase in these powerful regulators is thought to provide benefit to the newborn by dampening inflammatory responses to the establishing microbiome (64). However, the consequence of their increased frequency and activity is the potential for decreased virus-specific T cells in response to infection as an overly exuberant T_reg_ population can hamper generation of a sufficient number of effector T cells needed for viral clearance [e.g., (61, 65, 66)].

Antibody responses are also significantly decreased in neonates, with reported defects in the production of high level, high affinity antibody (41, 67). In humans IgG production is generally weak for the first year of life (45, 68). Although increased relative to IgG, IgM responses are also impaired as exemplified by RSV infection of human infants, where both IgM and IgG responses are poor (69). The altered CD4^+^ T cell differentiation is one likely contributor to antibody responses in newborns and young infants. Studies in newborn mice revealed IgG1 skewing, consistent with a Th2 biased response (70). Further, production of high affinity, isotype switched antibody in the germinal center relies on CD4^+^ T follicular helper cells (Tfh) (71–75). As with other arms of the T cell response, Tfh generation is compromised in newborns (76, 77). Tfh provide help to B cells through the production of cytokines, e.g., IL-21 together with IL-4, IFNγ, or IL-17, as well as through the expression of CD40L (78, 79). Their importance for influenza vaccine responses is supported by the strong correlation between Tfh and the development of memory B cell responses in humans administered the inactivated influenza vaccine (80).

Infants are also challenged by inherent defects in B cell survival and differentiation (81). A potential contributor to the latter is the reduced expression of BCMA and BAFF-R on neonate B cells (82). Engagement of BAFFR or BCMA on B cells promotes survival through upregulation of anti-apoptotic bcl-2 family members together with downregulation of the pro-apoptotic factors bim and bad (83). Following differentiation, plasmablast survival and differentiation into long-lived antibody secreting cells depends on APRIL, the expression of which is decreased in stromal cells that reside in the bone marrow of neonates, likely hampering the sustained presence of these cells (84, 85). Thus, B cells encounter hurdles all along the pathway of activation, differentiation, and survival that make it harder to generate and sustain long-lived, protective antibody.

## The Newborn Immune Response to Influenza Virus Infection

Our mechanistic understanding of the newborn response to influenza virus infection comes predominantly from analyses in mice. The earliest report of the increased disease in neonates using the mouse model comes from a study by Smith and colleagues showing newborn mice (1 day old) exhibited higher viral load and increased mortality following infection (86). Subsequent studies have made significant headway in uncovering the basis of the decreased control of influenza virus infection in this model (87–89). In a study by You et al., 7-day-old mice infected with the mouse adapted A/PR/8/34 (H1N1) (PR8) virus had a highly reduced (6-fold) IFNɤ^+^ CD8^+^ T cell response in the lungs compared to animals infected at 4 weeks of age (88). A decrease was also observed in the CD4^+^ compartment, albeit less than that observed for CD8^+^ cells. Interestingly, there was no evidence of an increased IL-4^+^ T cell response in the lungs of infant mice in this study. In addition a reduction in virus-specific T cells, the authors found that these effectors were reduced in their ability to clear virus as demonstrated by adoptive transfer of neonate vs. adult T cells (88). Increased susceptibility of newborn (here 2-day old) mice to influenza virus infection was also reported by Garvy and colleagues (87). This study focused on the capacity of T cells to enter into the lung and their movement to the airway. Newborns exhibited an approximately 4-day delay compared to their adult counterpart in entry of virus-specific T cells. In addition, these cells exhibited a distinct pattern of distribution in newborn mice, with a striking impairment in migration to the airway space. The failure of cells to migrate the airway correlated with a significant decrease in CXCL9 and CCL2. These data reveal that in addition to the challenges in generating an effector T cell response, newborns must also cope with difficulties in getting these effectors to the site of virus infection.

Recently, the overall transcriptional response in the lungs of influenza virus-infected 3-day-old mice was evaluated (89). The lungs of newborn pups showed transcriptional unresponsiveness following infection, with only a modest number of assessed genes differentially expressed as a result of influenza virus infection compared to their adult counterpart (4 vs. 55%). Consistent with known impairments of newborn cells discussed above, the top pathways impacted were those responding to pattern recognition receptors and dendritic cell maturation. Follow-up studies to further explore these results will undoubtedly increase our understanding of the influenza virus-specific response in the newborn.

It is well-established that antibody responses are also impaired in the context of respiratory infection of human newborns and young infants (67). With that said, there is surprisingly little information available on the antibody response generated in newborns and young infants following influenza virus infection, even for the mouse model. However, we can infer that a likely factor that will impact antibody is the reduced Tfh response in newborns (76, 77) and the limited expression of APRIL in newborn mice given the important role of this signal in the maintenance of antibody titers in the airways and serum following influenza virus infection of adult mice (90).

Although the mouse model is highly tractable, one of the limitations of this model is the difference in immune system development at birth compared to humans (41). The immune system of newborn mice does not reflect that of higher order animals until 5–7 days following birth. In addition, the rapid maturation of the immune system in mice further complicates the study of newborn immunity. The NHP affords a useful model to mitigate these limitations. The NHP lung is also more similar in structure to humans than is the mouse lung (91). Finally, there is a high degree of similarity between the NHP and humans in the distribution and responsiveness of TLR receptors (92), in contrast to mice (14). In our studies, influenza virus infection (PR8) of newborn (6–10-day old) NHP resulted in more pathology than adult NHP, even when the virus dose in the infants was decreased 5-fold (60). Somewhat surprisingly, the systemic IgG response in the two age groups at d14 p.i. was similar. Whether this is the case at later times as the antibody response continues to develop is not known. In contrast, analysis of antibody in the lung revealed a reduction in virus-specific IgG antibody as well as highly reduced BALT in the lungs of newborns (60). These data suggest a model wherein an impaired local immune response in the lungs of newborns contributes to the reduced clearance and increased disease observed following influenza virus infection.

We have also evaluated the epitope specificity of the antibody response following influenza virus infection in the newborn NHP model (93). The specificity of the antibody is an important determinant of response efficacy as there are epitope-dependent differences in clearance mechanisms and cross-strain reactivity (94–99). HA represents the major target of influenza-specific antibody (100). Five neutralizing sites have been described in the globular domain of this molecule (101). In adult mice, the relative representation of antibodies directed to these sites, i.e., their pattern of immunodominance (ID), is consistent within a given mouse strain, evolves over time, and is altered by the nature of the immunizing event (i.e., vaccination vs. infection) (102). Virtually nothing is known about age related alterations in antibody ID. The ID pattern established by infection is of particular interest in newborns given the potential effects of childhood vaccination on lifelong influenza A virus (IAV) immunity as evidence suggests that the first exposure to IAV antigens can mold the lifetime response (103). Our analysis revealed altered ID patterns in the early IgM anti-HA response in newborns vs. adults that converged over time. Somewhat surprisingly the ID profiles for IgG vs. IgA differed, suggesting isotype specific regulation of ID. This study also examined the generation of antibody capable of recognizing the conserved HA stem region, a target of universal vaccine approaches. These antibodies were readily generated in newborns; similar to what is observed in adults, they are subdominant to the HA head domain (93, 104).

**Figure 1:**
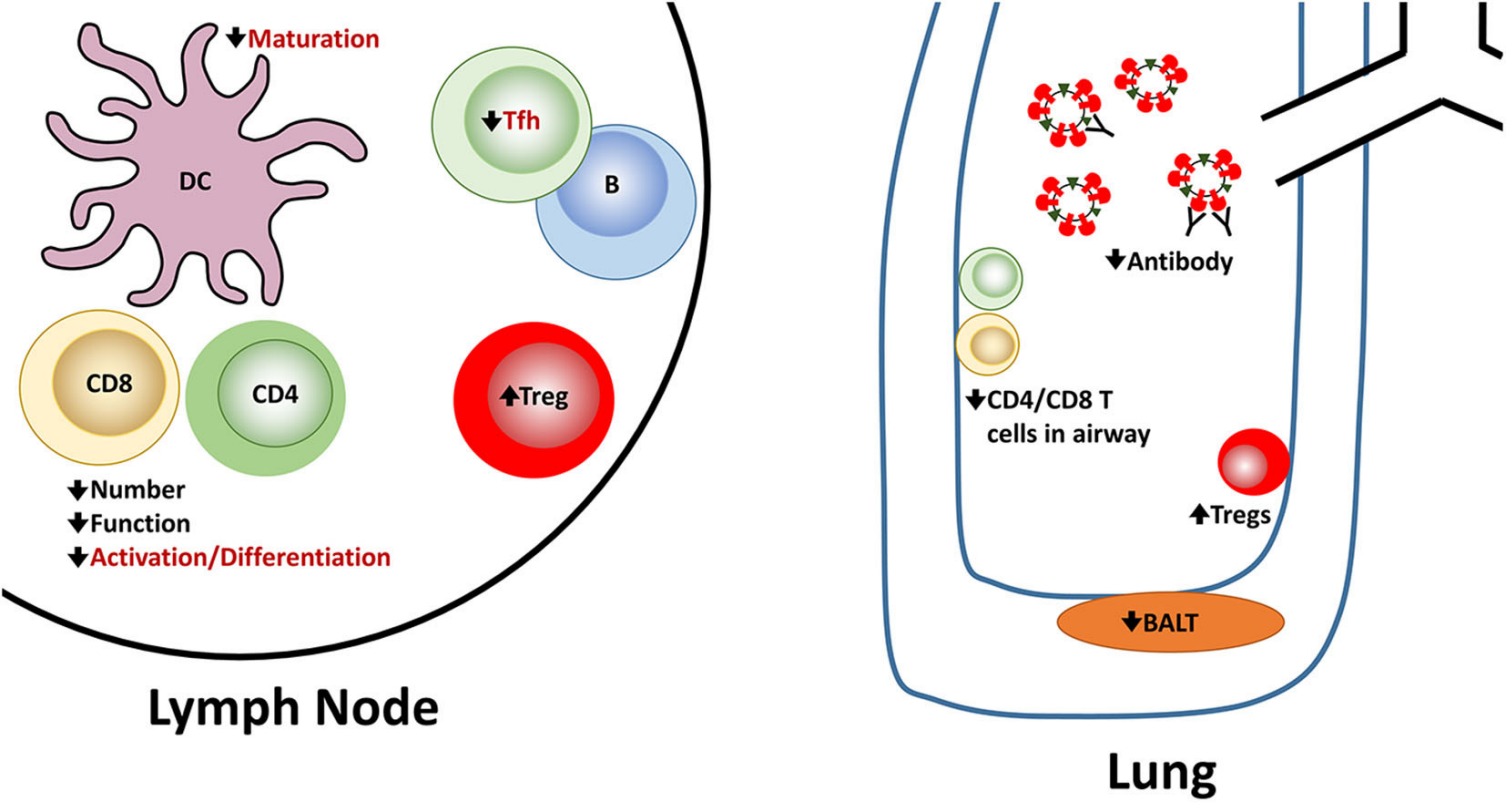
Reported and potential alterations in the newborn immune response following influenza virus infection. Newborns encounter challenges at multiple steps in the generation of an effective immune response following influenza virus infection. A number of these have been experimentally demonstrated in newborn animal models. These are indicated in black font. There are also alterations that seem highly likely in the context of influenza virus infection based on *in vitro* studies or other models of newborn responses as described in the text. However, they have not been directly shown for influenza virus infection of newborns. These proposed alterations are shown in red font.

In summary, newborns face multiple challenges in combatting infection with influenza virus (Figure 1). Both the innate and adaptive arms of the response exhibit alterations that make clearance less robust. Hurdles are present at the activation/differentiation phase that occurs in the lymph node as well as the effector stage in the lung, a site where the immune system must be able to clear virus efficiently while minimizing tissue damage.

## Influenza Vaccination to Protect Newborns

The ability to protect newborns from influenza infection through vaccination would represent a significant step forward in improving the health of this population. In considering how to harness the power of vaccines to maximize protection of newborns, there are two complementary approaches that can contribute to this goal, protection through passive transfer of vaccine-elicited maternal antibody and direct vaccination of the newborn.

Influenza vaccination for all pregnant women is the current recommendation and there is clear evidence of benefit to both mother and infant. Maternal vaccination has been reported to reduce proven influenza illness by 63% in infants up to 6 months of age and averted approximately a third of all febrile respiratory illnesses in mothers and young infants (105). Further, infants of vaccinated mothers were 45–48% less likely to have influenza hospitalizations than infants born to non-vaccinated mothers (106). Given these results, why should we not rely solely on this approach? While clearly providing increased protection for infants, the benefit of maternal antibody, as noted above, begins to wane after the first 8 weeks of life. For example, the efficacy of the trivalent inactivated influenza vaccine (TIV) administered during pregnancy for preventing PCR–confirmed influenza infection in the infants was 86% during the first 8 weeks, but decreased to 49% if considering the overall 6-month follow-up period (107). Another study reported that while ~40% of infants had protective antibody levels at birth, by 3 months this had declined drastically, with only 10% retaining protective levels (108). These results make evident the critical need to develop vaccines that can initiate protective antibody responses in young infants before the loss of protection that occurs as maternal antibody wanes.

Ideally, an influenza vaccine for newborns would be capable of inducing protective responses that would be in place following a single dose of vaccine, although admittedly this is a challenging goal given the altered immune system of newborns. Given the time required for development of the peak response and the likely need for a second dose of vaccine to induce protective antibody levels, as is the case even when the current vaccine is given at 6 months of age, the initial vaccination would need to occur within the first 2 months of life in order to minimize or eliminate a window of susceptibility.

This highlights the need for improved vaccine approaches for newborns, given that these individuals do not respond effectively to the current seasonal inactivated vaccine before the age of 6 months. Previous studies reported the virtual absence of seroconversion in infants between 3 and 5 months old after a single administration of the trivalent inactivated influenza vaccine with the exception of one virus strain (A/Mississippi/11/85), which had a 40% conversion rate for reasons that are unknown (109). While maternal antibody has the potential to inhibit vaccine responses (110), this was not the case here, as non-responding infants had influenza-specific antibody titers of <1:8. A second dose resulted in a protective titer rate of approximately 29% across all strains evaluated. Not surprisingly, a correlation was observed between age and seroconversion, with older infants converting at a higher rate than younger infants (109). In a second study, conversion was assessed following two doses of vaccine, with a reported conversion rate of 32% for H1N1 and 47% for H3N2 strains (110). Mechanistic studies were not performed in these trials, so how these infants responded at the level of T and B cell activation and differentiation is not known. A review of the literature reveals a surprisingly limited amount of information in animal models to inform our mechanistic understanding of the response to the seasonal inactivated influenza vaccine in newborns vs. adults.

Given these challenges, how do we achieve the objective of protecting newborns through vaccination? One area that remains an ever present goal for increasing the efficacy of vaccines is the development of new adjuvants. Intense effort has focused on exploiting the immune stimulatory properties of TLR agonists as adjuvants, including in the infant. Among the most promising are TLR7/8 agonists. The natural ligands of TLR7 and TLR8 are guanosine- and uridine-rich ssRNA (111, 112). However, a number of small molecule mimetics have been developed that are potential vaccine adjuvants. The TLR8 agonist 3M-002 induces potent upregulation of CD40, CD80, CD83 and CD86 as well as production of the Th1-polarizing cytokine IL-12p70 in cells from neonates (113). A contributor to the effectiveness of TLR8 agonists in the context of neonate cells appears to be the resistance of this pathway to inhibition by adenosine (113), a known suppressive immune modulator in the blood of newborns (114). We have found that an inactivated vaccine comprised of the TLR7/8 agonist R848 conjugated to the influenza virion is a potent inducer of antibody and IFNγ-producing T cell responses in a newborn NHP model (115, 116). TLR2 agonists also show promise in their ability to increase activation of newborn T cells (117). Further, select TLR ligands can induce maturation of newborn APC, approaching the level observed in adults (27, 113, 118). The TLR5 agonist flagellin has shown particular promise as a mucosal adjuvant for neonates in its ability to effectively induce maturation and migration of newborn lung-resident DC (27).

A strategy for further increasing the efficacy of these immune modulators is the delivery of multiple TLR agonists. Simultaneous engagement of several TLR has been shown to change dendritic cell maturation in both a qualitative and quantitative fashion (18, 119). T cells derived from human cord blood stimulated concurrently with TLR2 and TLR5 agonists underwent greater proliferation and cytokine production compared to cells stimulated with either agonist alone (117). The success of the tuberculosis vaccine (BCG: Bacille Calmette-Guerin), which is routinely delivered within 48 h of birth, supports the utility of this strategy. BCG contains ligands for 5 distinct TLR (1, 2, 4, 6, and 9) (120) and it seems likely that the ability to induce immune responses in neonates may be due to the combined signaling following engagement of multiple TLR.

In addition to TLR agonists, other approaches are also exhibiting potential in newborns. A recent study from Hensley and colleagues reported that a nucleoside modified mRNA-lipid nanoparticle vaccine was capable of inducing prolonged germinal center formation newborns (121). The efficacy of this adjuvant has been associated with high antigen production that drives Tfh and GC B cell responses (122). Interestingly, this approach could also partially mitigate the inhibitory effects of maternal antibody (121).

Vaccination of 1 week old mice with trehalose-6,6-dibehenate (TDB), a synthetic analog of the mycobacterially-expressed trehalose-6,6-dimycolate (TDM), in combination with HA in liposomes was found to induce protection of newborn mice through increases in Tfh and generation of high affinity plasma cells (123). This is the result of signaling that is induced following binding to the C-type lectin receptor Mincle. Ligands for this PAMP detecting molecule are primarily bacterially glycolipids (124). The squalene based adjuvant MF59 has also been reported to be effective in mouse, NHP, and human neonates (125–127). Newborn (1 week old) mice vaccinated with HA and MF59 had increased influenza-specific IgG, DC maturation, and CD4^+^ T cell responses. However, a potential limitation of MF59 is its failure to drive Tfh in the neonates.

**Figure 2:**
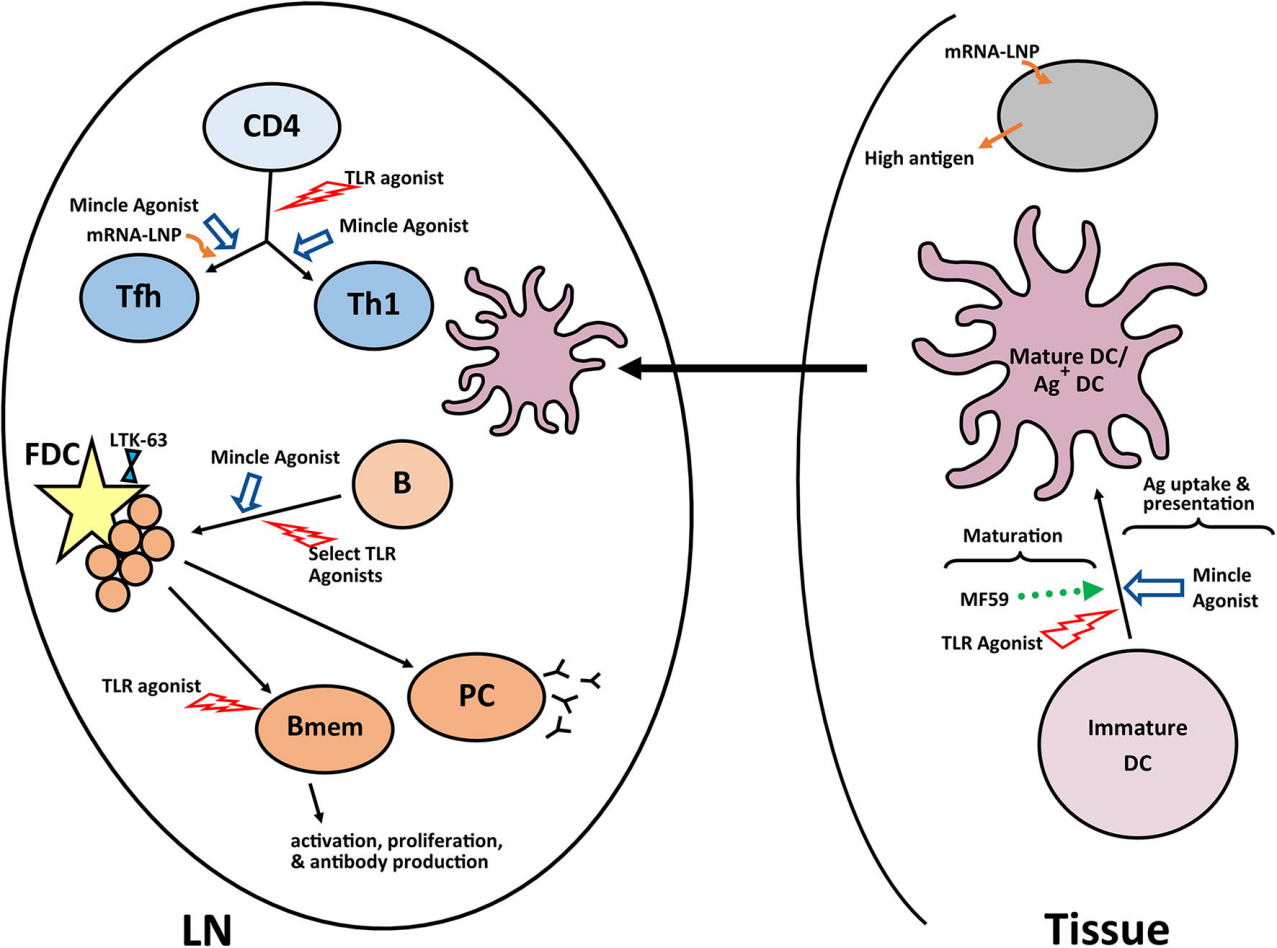
Adjuvants and pathways reported to promote responses in newborns. A number of experimental adjuvants have been tested in the context of neonates or neonate derived cells. Some of these adjuvants impact multiple cell types, while others are more targeted. The results from these analyses suggest combinations of adjuvants may be needed to overcome the multiple impairments in the newborn immune response. A summary of a representative selection of promising adjuvants for newborns is shown. LN, lymph node; GC, germinal center; PC, plasma cell; Bmem, memory B cell; DC, dendritic cell; FDC, follicular dendritic cell.

The development of a robust germinal center is dependent on development of a mature follicular dendritic cell network, which occurs inefficiently in newborn mice (128). Using a tetanus toxoid conjugated polysaccharide vaccine, Jonsdottir and colleagues showed a non-toxic mutant of *Escherichia coli* heat-labile enterotoxin (LT-K63) could drive the maturation of the FDC network in newborn mice and that this resulted in the increased number and prolonged survival of IgG^+^ antibody secreting cells (129). While exciting, there is recent evidence that the effects of this adjuvant can vary depending on antigen, route of delivery, and mouse strain (130). This is likely true for many of the adjuvants discussed suggesting a caution and making clear the need for empirical testing of each vaccine construct with the experimental adjuvant. A summary of the pathways successfully targeted by adjuvants in newborns is shown Figure 2.

## Vaccination as the First Exposure to Influenza Antigens

As noted above, infants cannot receive their first dose of the seasonal inactivated influenza vaccine until they reach 6 months of age. This allows for infection to serve as the first exposure of many infants to influenza antigens. Although the priming environment for immune activation includes all of the stimulatory signals associated with infection, it comes at the cost of the potential for severe disease as described above. If we realize the goal of producing an effective influenza vaccine for use in newborns, it would become likely that the first antigen exposure for this at-risk population would come through vaccination.

A number of landmark studies have firmly established the contribution of antigenic seniority as a governing factor in driving immune responses to influenza vaccination or infection [for review see (131)]. The notion of antigenic seniority states that strains encountered early in childhood establish a position of seniority, such that these responses are preferentially boosted following encounter with alternative strains over a lifetime. This then shapes an individual's response to a given strain confronted later in life and over time, there is an accumulation of antibody and memory B cells specific for childhood strains.

Adding an additional layer of complexity to this process, in mice there is evidence that the immune response primed by vaccination vs. infection is altered (102). Using a panel of viruses that individually expresses each of the five neutralizing epitopes identified in the HA head, Angeletti et al., showed that the pattern of immunodominance, i.e., the representation of each of the HA epitopes in the antibody repertoire, was distinct following infection vs. vaccination (102). This has the potential to functionally impact the response as the representation of antibodies directed to individual epitopes can impact how the virus is cleared, i.e., neutralization vs. antibody dependent cellular cytotoxicity (ADCC). While, the ability of vaccination vs. infection to alter dominance patterns in newborns has not been tested, the results suggest an intriguing possibility that newborn priming of influenza-specific responses by vaccination vs. infection may result in differences in the representation of antibodies to these individual epitopes. This may in turn alter the array of mechanisms available for combating infection as well as the specificity of antibodies boosted following subsequent encounter with viruses or vaccines in the following years.

The concept of antigenic seniority has taken on added importance in newborn vaccination in light of recent efforts directed toward development of a universal vaccine approach. A vaccine that can protect from drifted strains, i.e., those that have point mutations in the globular head domain of the HA molecule, that circulate in subsequent seasons would decrease the need for yearly reformulation and delivery of the vaccine. Such a vaccine also has the potential to provide protection from pandemic strains. Currently universal vaccine development is primarily focused on driving antibody responses to the highly conserved stem region of HA [there are multiple excellent reviews on this effort, e.g., (95, 132–134)]. Such a vaccine is now being tested in adults in clinical trials (NCT03814720, www.clinicaltrials.gov).

**Figure 3:**
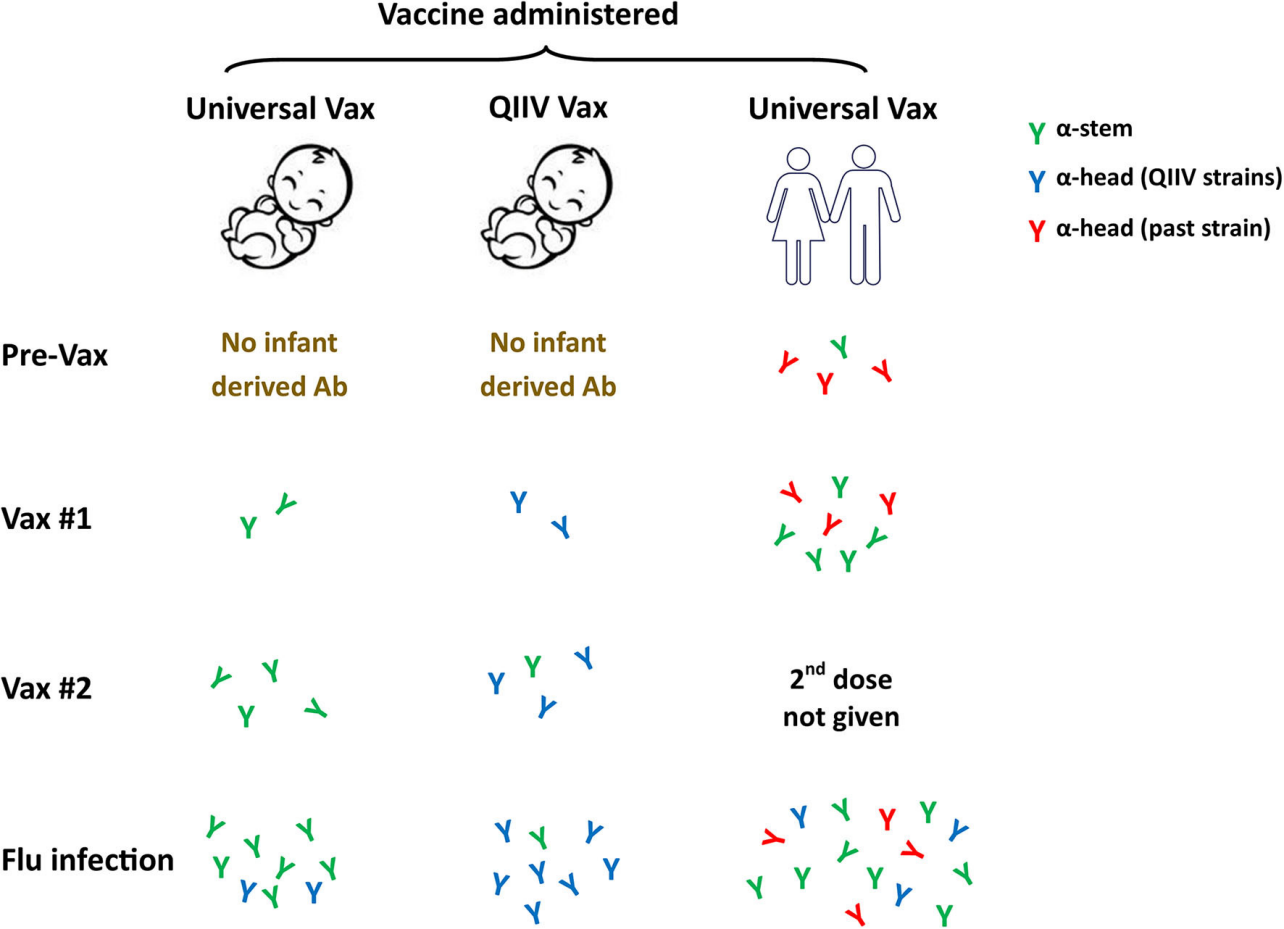
Universal influenza vaccines as a first antigen exposure in newborns. Current influenza vaccine efforts are focused on the development of approaches that can provide broad strain recognition, i.e., a universal vaccine. These vaccines often target the conserved stem region of the HA molecule. The ability to successfully employ a universal vaccine that can work in newborns through appropriate adjuvantation would likely result in HA stem as the first influenza antigen exposure. Subsequent virus challenge should preferentially drive these universal specificities and may result in their higher representation compared to what would occur in individuals who received the stem vaccine later in life. Whether this single specificity response, e.g., stem-specific antibody, will provide adequate protection will need to be carefully assessed. QIIV, quadrivalent inactivated influenza vaccine.

Is this approach feasible in newborns? The answer may be yes based on our recent studies of newborn NHP in which we observed a robust response to the HA stem region following infection (93). These data suggest the newborn B cell repertoire has the potential mount this response. Further, the avidity of the HA stem-specific antibodies produced appears to be on par with antibodies binding to other epitopes (93). Our understanding of antigenic seniority would indicate that priming the newborn response with an HA stem construct would be of significant benefit. Pre-clinical studies performed in adult mice and ferrets vaccinated with a stem bearing nanoparticle construct resulted in generation of broadly cross-reactive antibodies (135). These antibodies could completely protect mice and partially protect ferrets against a lethal heterosubtypic H5N1 influenza virus challenge (135). We would expect that antibody responses generated by an HA stem vaccine would allow for preferential activation and boosting of these broadly protective antibody responses following infection or subsequent vaccination (Figure 3). The response will likely differ in human newborns vs. adults, as the latter already has a population of memory cells that can respond to the vaccine that will undergo selective boosting. Priming responses from a naïve repertoire in the newborn would leave the infant with a single antibody specificity. While stem-specific antibodies should provide benefit through broad strain recognition, it is critical that we understand more fully the protective capacity of such a vaccine. In addition, how this universal response would impact subsequent responses to the head region of HA or other viral proteins needs to be addressed. Answering these questions will be a critical step in advancing universal vaccines for use in infants.

## Concluding Remarks

Newborn vulnerability to severe disease following influenza virus infection is a significant public health concern. Current influenza vaccines are inadequate for eliciting protective responses in these at-risk individuals and while maternal antibody can provide benefit, waning antibody levels will leave the infant increasingly unprotected in the months following birth. The time necessary for a prime and boost-approach to achieve protective antibody levels necessitates early delivery of the first vaccine dose to limit the window of susceptibility to infection that will occur as maternal antibody decreases and infant immunity develops. While a number of experimental adjuvants show promise in newborns, a more in depth understanding of the newborn immune system will be critical to the development of effective adjuvants and vaccine delivery approaches. This will be facilitated by use of models that most faithfully reflect the human newborn immune system coupled with our increasing ability to assess human newborns as a result of technological advances that maximize the information that can be gained with the limited samples accessible from these individuals. While the challenges are significant, they are not insurmountable, and we are continually nearing the goal of developing a vaccine that can limit influenza virus infection and disease in newborns.

## Author Contributions

MA-M wrote and edited the manuscript.

## Conflict of Interest

The author declares that the research was conducted in the absence of any commercial or financial relationships that could be construed as a potential conflict of interest.
